# Causal Effects of Homocysteine, Folate, and Cobalamin on Kidney Function: A Mendelian Randomization Study

**DOI:** 10.3390/nu13030906

**Published:** 2021-03-11

**Authors:** Sehoon Park, Soojin Lee, Yaerim Kim, Semin Cho, Kwangsoo Kim, Yong Chul Kim, Seung Seok Han, Hajeong Lee, Jung Pyo Lee, Kwon Wook Joo, Chun Soo Lim, Yon Su Kim, Dong Ki Kim

**Affiliations:** 1 Department of Biomedical Sciences, Seoul National University College of Medicine, Seoul 03080, Korea; mailofsehoon@gmail.com (S.P.); yonsukim@snu.ac.kr (Y.S.K.); 2 Department of Internal Medicine, Armed Forces Capital Hospital, Gyeonggi-do 13574, Korea; 3 Department of Internal Medicine, Seoul National University Hospital, Seoul 03080, Korea; sjlee891016@hanmail.net (S.L.); jseminy@gmail.com (S.C.); imyongkim@gmail.com (Y.C.K.); hansway80@gmail.com (S.S.H.); mdhjlee@gmail.com (H.L.); nephrolee@gmail.com (J.P.L.); junephro@gmail.com (K.W.J.); cslimjy@snu.ac.kr (C.S.L.); 4 Department of Internal Medicine, Seoul National University College of Medicine, Seoul 03080, Korea; 5 Department of Internal Medicine, Keimyung University School of Medicine, Daegu 42601, Korea; yaerim86@gmail.com; 6 Transdisciplinary Department of Medicine & Advanced Technology, Seoul National University Hospital, Seoul 03080, Korea; kksoo716@gmail.com; 7 Kidney Research Institute, Seoul National University, Seoul 08826, Korea; 8 Department of Internal Medicine, Seoul National University Boramae Medical Center, Seoul 07061, Korea

**Keywords:** mendelian randomization, homocysteine, folate, cobalamin, chronic kidney disease

## Abstract

Blood homocysteine level and related vitamin levels are associated with various health outcomes. We aimed to assess causal effects of blood homocysteine, folate, and cobalamin on kidney function in the general population by performing Mendelian randomization (MR) analysis. Genetic instruments for blood homocysteine, folate, and cobalamin levels were introduced from a previous genome-wide association (GWAS) meta-analysis of European individuals. Summary-level MR analysis was performed for the estimated glomerular filtration rate (eGFR) from the CKDGen consortium GWAS that included 567,460 European ancestry individuals. For replication, allele-score-based MR was performed with an independent U.K. Biobank cohort of 337,138 individuals of white British ancestry. In summary-level MR for the CKDGen data, high genetically predicted homocysteine levels were significantly associated with low eGFR (per 1 standard deviation, beta for eGFR change −0.95 (−1.21, −0.69) %), supported by pleiotropy-robust MR sensitivity analysis. Genetically predicted high folate levels were significantly associated with high eGFR change (0.86 (0.30, 1.42) %); however, causal estimates from cobalamin were nonsignificant (−0.11 (−0.33, 0.11) %). In the U.K. Biobank data, the results were consistently identified. Therefore, a high blood homocysteine level causally decreases eGFR. Future trials with appropriate homocysteine-lowering interventions may be helpful for the primary prevention of kidney function impairment.

## 1. Introduction

Chronic kidney disease (CKD) is a major comorbidity associated with a large socioeconomic burden and risk of mortality [1]. The prevalence of kidney function impairment is increasing along with the global aging trend and the growing population with obesity. Appropriate lifestyle modifications and risk factor management, including the control of metabolic disorders, have been recommended to maintain healthy kidney function in the general population.

Hyperhomocysteinemia has been suggested to be associated with various adverse health outcomes [2,3]. High homocysteine levels are related to risks of cardiovascular diseases or kidney function impairment, particularly in populations with kidney function impairment [4,5,6]. However, as observational findings have been conflicted and kidney function itself determines blood homocysteine levels [7,8], whether a high blood level of homocysteine is a causative factor for kidney function impairment has yet to be determined. A previous randomized clinical trial showed that supplementation with folate, which decreases homocysteine levels, resulted in delays in CKD progression and stroke in a Chinese population where folate food fortification is not mandatory [9,10]. However, the effects of homocysteine-lowering therapy showed limited efficacy for secondary prevention of cardiovascular diseases or kidney dysfunction, particularly in countries with folate fortification policies [11,12]. Given the conflicting results, additional studies investigating the causal effects of blood homocysteine levels on kidney function parameters are warranted, but the observational findings are prone to reverse causation or effects from confounders.

Mendelian randomization (MR) is an analytic tool for investigating the effects of a modifiable risk factor on complex diseases [13]. As the instrumented genotype is fixed before birth, the causal estimates from MR are minimally affected by reverse causation or confounding effects. Previous MR analyses have reported important causal effects of various serum biomarkers or environmental factors predicted by genetic instruments on health outcomes [14,15].

In this study, we aimed to investigate the causal effects of homocysteine and vitamin Bs in the homocysteine metabolic pathway, including folate (vitamin B9) and cobalamin (vitamin B12), on the estimated glomerular filtration rate (eGFR) by MR analysis. We studied the causal effects of blood biomarkers on kidney function in the two largest genotyped datasets for kidney function traits to date. We mainly hypothesized that high genetically predicted homocysteine levels would be causally linked to low kidney function in the general population.

## 2. Materials and Methods

### 2.1. Ethics Approval

The study was performed in accordance with the Declaration of Helsinki. The study was approved by the Institutional Review Boards of Seoul National University Hospital (No. E-2070-048-1140) and the U.K. Biobank consortium (application No. 53799). As the study investigated anonymous databases or summary-level data, the requirement for informed consent was waived by the institutional review boards.

### 2.2. Study Setting

**Figure 1:**
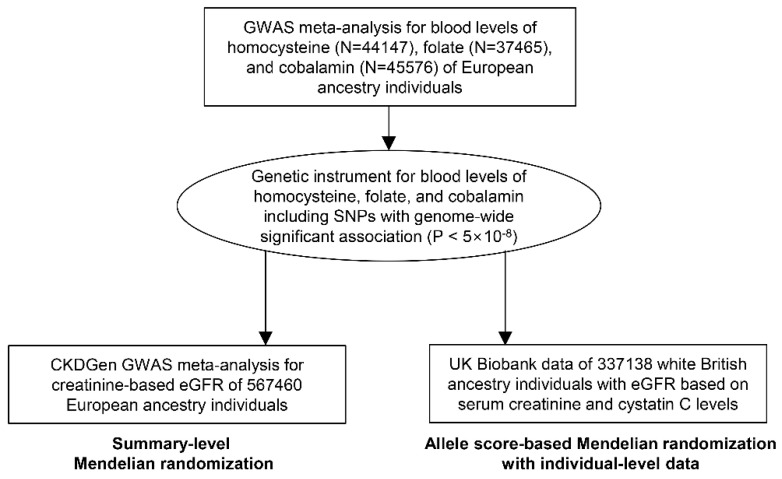
Study flow diagram.

This study was an MR analysis including outcome assessment in two population-scale datasets (Figure 1). Genetic instruments for blood homocysteine, folate, and cobalamin levels were introduced from a previous genome-wide association study (GWAS) analyses [16,17]. First, the genetic instrument was applied to the summary-level MR, and the outcome summary statistics for a kidney function marker, eGFR, were provided by the CKDGen consortium, which is the largest in scale to date [18]. Second, the MR analysis was performed with individual-level data from the U.K. Biobank for replication.

### 2.3. Genetic Instrument

The study utilized the previous GWAS meta-analysis results for blood total homocysteine, folate, and cobalamin levels [16,17]. The single nucleotide polymorphisms (SNPs) reaching the genome-wide significance threshold (*p* < 5 × 10^−8^) for the exposure phenotypes were selected from the previous GWAS, which included 44,147, 37,465, and 45,576 individuals of European ancestry for total homocysteine, folate, and cobalamin levels, respectively [16,17]. The utilized SNPs were not in linkage disequilibrium (r^2^ < 0.1 in the 1000 G European population). The summary statistics of the genetic instrument for blood total homocysteine (N of SNPs = 18), folate (N of SNPs = 3), and cobalamin levels (N of SNPs = 14) are presented in Table 1. As the studied exposure biomarkers are in a metabolic pathway, genetic correlation between the instruments for each studied biomarker was identified; 1 SNP on the methylenetetrahydrofolate reductase (*MTHFR*) gene overlapped, and another SNP was in linkage disequilibrium between the genetic instrument for homocysteine and folate level. Similarly, 4 SNPs of the genetic instrument for homocysteine level were correlated with the 4 SNPs predicting cobalamin levels.

### 2.4. Considerations for Key Assumption of MR

The MR analysis requires that three assumptions be met to demonstrate causal effects [13]. First, the relevance assumption is that the genetic instrument should be strongly associated with the exposure of interest, and the assumption was met by utilizing the SNPs reaching genome-wide significant level association. Second, the independence association is that the genetic instrument should not be associated with confounders. We performed well-known MR sensitivity analysis available for summary-level MR, relaxing this assumption for some of the instruments [19,20]. Third, the exclusion-restriction assumption is that the causal effects should be through the exposure of interest and cannot be formally tested. However, median-based sensitivity MR methods relax this assumption in up to 50% of the genetic instruments and are thus considered a sensitivity analysis for this assumption [20]. Further, we tested the causal effects from a single variant that is biologically proven for its effect on the homocysteine metabolic pathway and the causal estimates from the single-variant MR, which would be minimally biased from a horizontal pleiotropic pathway.

### 2.5. Summary-Level MR with the CKDGen Data

As the genetic instruments were from GWAS results of individuals of European ancestry, the CKDGen data for log-transformed eGFR, determined by serum creatinine levels, of 567,460 individuals of European ancestry were downloaded from the public repository (URL: https://ckdgen.imbi.uni-freiburg.de/, last accessed date 7 March 2021) [18]. The study meta-analyzed 85 GWAS results; the population had a median age of 50.1 years with 48% males, and the median eGFR value was 91.4 mL/min/1.73 m^2^.

In the summary-level MR, the SNPs that were nonoverlapping were excluded during the harmonization of the summary statistics [21]. To ensure that the effects were from genetic predisposition for the studied biomarkers to eGFR, rather than the reverse direction, Steiger filtering was performed as previously described [22].

The main MR method was the conventional fixed-effects inverse variance weighted method. As the inverse variance weighted method can be biased from a pleiotropic effect, additional sensitivity MR analysis is necessary to test the attainment of the MR assumptions. First, MR-Egger regression with bootstrapped standard errors was performed, and MR-Egger regression provides pleiotropy-robust causal estimates [19]. Additionally, the MR-Egger intercept *p* values were calculated, which is the formal test for detecting the presence of directional pleiotropy. Second, the penalized weighted median method was implemented, which derives valid causal estimates even under conditions when invalid instruments are present [20]. The median-based method relaxes the independence and the exclusion-restriction assumption for up to 50% of the instrumented weights. Third, we performed the contamination mixture method, which detects groups of genetic variants with similar causal estimates and performs robust MR analysis in the presence of invalid instruments [23].

In a separate analysis, we instrumented a single variant (rs1801133), which is on the *MTHFR* gene, which codes the enzyme involved in homocysteine–folate metabolism. Implementing a single biologically proven variant has advantages, as the analysis would give pleiotropy-robust results, although the instrumental power generally decreases by limiting the number of SNPs included in the genetic instrument. The SNP has been well studied for its effect on homocysteine levels and health outcomes mediated by blood homocysteine or folate levels [24]. The SNP showed the strongest association strength with blood homocysteine level in the currently implemented GWAS meta-analysis (*p* = 4.34 × 10^−104^). As the effect from the single SNP would hardly be from a horizontal pleiotropic pathway, the causal estimates from the single variant would certainly attain the key MR assumptions. In this analysis, the Wald ratio method was the MR analysis tool used to yield causal estimates.

The effect sizes were transformed to a % change in eGFR units. The summary-level MR analysis was performed by the TwoSampleMR package in R (version 4.0.2, the R foundation) [25], and two-sided *p* values <0.05 were considered significant.

### 2.6. Allele-Score-Based MR with Individual-Level Data in the U.K. Biobank

The U.K. Biobank is a prospective population-based cohort of >500,000 individuals aged 40–69 years collected from 2006 to 2010 in the United Kingdom [26,27]. The data have strength for our MR analysis, as the U.K. Biobank cohort is independent from the CKDGen data. Thus, the analysis with the data would be an independent replication of the summary-level MR results. In addition, as the data are phenotyped for multiple clinical characteristics, adjustments for important comorbidities related to kidney function were possible. The limitation of the data is that healthy volunteer bias is present, and the U.K. Biobank participants had lower prevalence of CKD than the general population [28].

For the analysis, we included the U.K. Biobank data of unrelated individuals of white British ancestry. Those who were outliers in terms of heterozygosity or missing rate and those with sex chromosome aneuploidy were excluded, resulting in 337,138 individuals assessed for allele-score-based MR [15]. The median age was 58 years, and 46% were males; the median eGFR was 92.5 mL/min/1.73 m^2^ determined by the Chronic Kidney Disease Epidemiology Collaboration (CKD-EPI) equation based on serum creatinine levels.

We calculated allele scores for the exposures by multiplying the gene dosage matrix with the effect sizes of the genetic instrument by using PLINK 2.0 (version alpha 2.3) [29]. The associations between the allele scores and eGFR were investigated by linear regression analysis, adjusted for age, sex, genotype measurement batch, and the first 10 principal components. Additional sensitivity analysis was performed, including hypertension, diabetes mellitus, and obesity as the covariates in the regression model.

## 3. Results

### 3.1. Summary-Level MR Results

In the summary-level MR, 16 genetic variants remained in the genetic instrument for homocysteine, as two SNPs were excluded due to nonoverlapping. The genetic instrument for folate included three SNPs, as none of them were disregarded during the harmonization process. There were 12 SNPs remaining as the genetic instrument for cobalamin as two SNPs were disregarded due to nonoverlapping. Steiger filtering showed that the causal effects from the genetic variants were from exposures to outcome, and none of the SNPs were disregarded by the filtering process.

**Figure 2:**
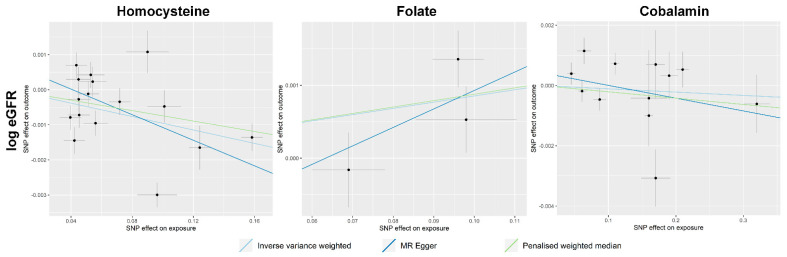
Scatter plots showing the results for summary-level Mendelian randomization analysis results. The x-axes indicate the effect for exposure variables, and the y-axes indicate the effect for outcome (log-transformed eGFR). The analysis was performed for 567,460 individuals of European ancestry of the CKDGen consortium.

The scatter plots demonstrating the summary-level MR results are presented in Figure 2. The causal estimates demonstrate that high total homocysteine levels were causally linked to low eGFR values (Table 2). The results were significant in both the MR-Egger and penalized weighted median methods, and the MR-Egger intercept *p* value indicates the absence of significant directional pleiotropy. On the other hand, high genetically predicted folate levels were significantly associated with high eGFR values, and the findings were consistent throughout the utilized MR sensitivity analyses. However, genetically predicted cobalamin levels were nonsignificantly associated with eGFR outcomes in the CKDGen data.

When the single SNP rs1801133 was implemented as the genetic instrument, the homocysteine-increasing genetic predisposition was significantly associated with lower eGFR (per 1 standard deviation increase in the genetic predisposition, eGFR change −0.85 (95% confidence interval −1.33; −0.38) %.

### 3.2. Allele-Score-Based MR Results

A high allele score for blood total homocysteine level was significantly associated with low eGFR (Table 3). Even after phenotypical hypertension, diabetes mellitus, and obesity status were additionally adjusted, the results remained significant. On the other hand, a high allele score for folate level was significantly associated with high eGFR values. The results were similar when the phenotypical covariates were adjusted. The allele score for cobalamin level was nonsignificantly associated with the outcomes, similar to the summary-level MR results.

## 4. Discussion

In this MR analysis, we identified that a high total blood homocysteine level was causally linked to low eGFR in the summary-level MR with the largest genetic dataset to date for eGFR provided by the CKDGen consortium. The findings were consistent for pleiotropy-robust MR sensitivity analysis, and the results were replicated by independent population-scale data from the U.K. Biobank. Therefore, this study supports that a high blood homocysteine level is a causative factor for reduced kidney function.

Observational associations between blood homocysteine levels and kidney function or cardiovascular disease have been repeatedly reported. However, the blood level of a biomarker can be increased due to impaired kidney clearance (e.g., reverse causation), and whether blood homocysteine levels can cause decreased kidney function cannot be answered by previous observational findings. A previous subanalysis of a randomized trial including Chinese individuals reported that folate supplementation, with a drop in blood homocysteine levels, can delay the progression of kidney function impairment [10]. However, the study was limited to an Asian ethnic population, and a replicative finding for kidney function outcome has rarely been reported. Additionally, conflicting results with composite vitamin B supplementation were reported in Canadian diabetic kidney disease patients [12]. Therefore, additional studies investigating the causal effects of blood homocysteine levels on kidney function are warranted, and in this study, this was performed through MR analysis, which has the strength to identify causal estimates from a genetically predictable biomarker. Through our efforts to attain the MR assumptions and as the direction of the genetic effect was inspected, the consistent findings of our MR analysis support that a high blood homocysteine level causally decreases kidney function. Our study has strength, as the study findings were replicated in two population-scale datasets and included individuals of European ancestry in whom the effects of homocysteine on kidney function had yet to be reported. Furthermore, the study population was not limited to those with advanced CKD, and most of the studied individuals had preserved eGFR; thus, the adverse effects of blood homocysteine on kidney function may be present in the general population.

Homocysteine is a nonprotein amino acid present in the methionine metabolism pathway. In this pathway, folate is activated by the MTHFR enzyme, which converts 5,10-methylenetetrahydrofolate to 5-methyletetrahydrofolate, which is necessary for converting homocysteine to another amino acid. High concentrations of blood homocysteine have been reported to cause pathologic vascular changes [30]. Homocysteine binds to proteins and modifies their function through the so-called homocysteinylation process, which occurs proportionally to the blood homocysteine level. When homocysteine binds with a thiol group, the redox status of a protein can be altered, further leading to oxidative stress injury [31]. Homocysteine can induce collagen synthesis, resulting in vascular endothelial dysfunction and stiffness [32]. In addition, homocysteine has been reported to be linked to inflammation, which may further contribute to the consequent facilitation of atherosclerotic injury [33]. Such effects of homocysteine on vascular injury may be the mechanism of the adverse causal effects of high blood homocysteine levels, supported by the MR analysis of small vessel stroke in a previous study and kidney function in the current analysis [14].

Based on our findings, a future clinical trial may target homocysteine-lowering therapy to prevent kidney function loss. As folic acid supplementation is the most well-recognized method of lowering blood homocysteine levels, such an intervention may initially be considered. The benefits of folate supplementation for the cardiovascular outcome of CKD patients have been suggested by a previous meta-analysis, particularly when homocysteine was effectively lowered [34]. However, some contradictory reports were present in the United States and Canada, and the efficacy of vitamin B supplementation to lower blood homocysteine levels was absent as the secondary prevention of cardiovascular diseases or diabetic kidney disease [11,12,35]. One of the previous studies addressed a nonvitamin homocysteine-lowering strategy that may be considered due to possible vitamin B toxicity in those with reduced kidney function who are already receiving a folate-fortified diet [12]. Therefore, although folate supplementation may be a worthwhile intervention for lowering blood homocysteine levels to prevent kidney function impairment, particularly in European or Asian countries without a folate fortification policy, other future strategies that effectively lower blood homocysteine levels may also be considered. Specifically, multiple vitamin B supplementation in populations receiving a folate-fortified diet may be discouraged, considering the possibility of a harmful effect of additive intake of vitamin B [12,35]. Furthermore, regarding the previous negative results in a population with mostly established kidney function impairment [12], if a folate supplement is being trialed, the intervention may target primary, rather than secondary, prevention of CKD in the general population without reduced kidney clearance. Those with a high blood homocysteine level or with folate deficiency may be the primary target group for such trials.

The current study has several limitations. First, the genetic effect sizes in MR analysis cannot directly reflect the potential clinical effect size of the related intervention [36]. The actual effects of the homocysteine-lowering strategy on kidney function should be answered in a future clinical trial, and the possibility that the efficacy may be different according to the trialed homocysteine-lowering intervention should be taken into account. Second, as the eGFR outcome was from those with relatively preserved kidney function, the effects of homocysteine levels in those with advanced CKD cannot be investigated herein. Thus, the study cannot advocate a trial for secondary prevention of CKD by homocysteine-lowering interventions, which was also discouraged by a previous clinical trial [12]. Third, the study was based on individuals of European ancestry; thus, the findings may not be generalizable to those with other ethnic backgrounds. Last, the study outcome estimated kidney function based on creatinine values, which may be affected by one’s body shape or diet. A future clinical study may consider including measured kidney function or more robust kidney function parameters. 

In conclusion, a high blood homocysteine level is causally linked to a low eGFR. A future clinical trial investigating the efficacy of homocysteine-lowering interventions is warranted for the primary prevention of kidney function impairment.

## Figures and Tables

**Table 1:** The summary statistics of the genetic instrument for blood total homocysteine (N of SNPs = 18), folate (N of SNPs = 3), and cobalamin levels (N of SNPs = 14).

Genetically Predicted Exposure	SNP	Effect Allele	Effect Allele Frequency	Beta	*p* Value
Homocysteine	rs1801133	A	0.34	0.1583	4.34E-104
	rs2275565	T	0.21	−0.0542	1.96E-10
	rs7422339	A	0.33	0.0864	4.58E-27
	rs9369898	A	0.62	0.0449	2.17E-10
	rs7130284	T	0.07	−0.1242	1.88E-20
	rs154657	A	0.47	0.0963	1.74E-43
	rs234709	T	0.45	−0.0718	3.90E-24
	rs4660306	T	0.33	0.0435	2.33E-09
	rs548987	C	0.13	0.0597	1.12E-08
	rs42648	A	0.4	−0.0395	1.97E-08
	rs1801222	A	0.34	0.0453	8.43E-10
	rs2251468	A	0.65	−0.0512	1.28E-12
	rs838133	A	0.45	0.0422	7.48E-09
	rs12134663	A	0.8	−0.101	2.54E-21
	rs12780845	A	0.65	0.0529	7.80E-10
	rs957140	A	0.45	−0.045	2.43E-10
	rs12921383	T	0.87	−0.09	8.22E-11
	rs2851391	T	0.47	0.056	1.70E-12
Folate	rs1801133	G	0.67	0.096	9.50E-53
	rs17421511	G	0.83	0.098	1.80E-15
	rs652197	C	0.18	0.069	1.20E-14
Cobalamin	rs602662	A	0.6	0.16	2.40E-139
	rs34324219	C	0.88	0.21	1.10E-111
	rs34528912	T	0.04	0.17	2.10E-15
	rs117456053	G	0.98	0.16	1.90E-09
	rs1801222	G	0.59	0.11	3.30E-75
	rs56077122	A	0.33	0.087	4.80E-21
	rs2336573	T	0.03	0.32	8.40E-59
	rs1131603	C	0.055	0.19	4.90E-49
	rs5753231	C	0.79	0.064	7.50E-10
	rs41281112	C	0.95	0.17	8.90E-35
	rs1141321	C	0.63	0.061	3.60E-26
	rs3742801	T	0.29	0.045	1.70E-13
	rs2270655	G	0.94	0.066	2.20E-13
	rs12272669	A	0.0022	0.51	3.00E-09

The genetic effect sizes are to reflect one standard deviation increase in the phenotype.

**Table 2:** Summary-level Mendelian randomization results with the CKDGen data.

Genetically Predicted Exposure	MR Method	MR-Egger Intercept *p* Value	Beta of eGFR Change (%) and 95% CI	*p* Value for Causal Estimate
Homocysteine	Inverse variance weighted	0.54	−0.95 (−1.21, −0.69)	<0.001
	MR-Egger		−1.81 (−2.59, −1.02)	<0.001
	Penalized weighted median		−0.74 (−1.15, −0.33)	<0.001
	Contamination mixture		−0.60 (−1.09, −0.20)	<0.001
Folate	Inverse variance weighted	0.51	0.86 (0.30, 1.42)	0.002
	MR-Egger		2.57 (0.70, 4.47)	0.001
	Penalized weighted median		0.88 (0.20, 1.56)	0.011
	Contamination mixture		0.90 (0.10, 1.61)	<0.001
Cobalamin	Inverse variance weighted	0.33	−0.11 (−0.33, 0.11)	0.333
	MR-Egger		−0.42 (−0.84, 0.00)	0.025
	Penalized weighted median		−0.21 (−0.59, 0.17)	0.276
	Contamination mixture		−0.10 (−0.40, 0.20)	0.092

MR = Mendelian randomization, CI = confidence interval; The analysis was performed with the summary statistics for log-transformed estimated glomerular filtration rate (eGFR) from the genome-wide association study meta-analysis conducted by the CKDGen consortium, which included 567,460 individuals of European ancestry. The effect sizes of the causal estimates were from a one standard deviation increase in the genetic predisposition for the plasma biomarkers towards eGFR change (%).

**Table 3:** Allele-score-based Mendelian randomization results with the U.K. Biobank data.

Outcome	Genetically Predicted Exposure	Main Analysis ^a^		Clinical Covariates Adjusted ^b^	
		Beta for eGFR (Continuous) and 95% CI	*p*	Beta for eGFR (Continuous) and 95% CI	*p*
eGFR (mL/min/1.73 m^2^)	Homocysteine	−0.108 (−0.148, −0.068)	1.6 × 10^−7^	−0.106 (−0.147, −0.066)	2.3 × 10^−7^
	Folate	0.067 (0.027, 0.108)	0.001	0.061 (0.021, 0.101)	0.003
	Cobalamin	0.005 (−0.035, 0.046)	0.802	0.004 (−0.037, 0.044)	0.855

eGFR = estimated glomerular filtration rate, CI = confidence interval; All allele scores were scaled to a one standard deviation increase; **^a^** The logistic regression model was adjusted for age, sex, genotype measurement batch, and the first 10 principal components of the genetic information. The analysis was performed in 337,138 individuals of white British ancestry in the U.K. Biobank cohort and a total of 321,260 individuals had creatinine-based eGFR values. **^b^** Phenotypical hypertension, diabetes mellitus, and obesity were added to the main model. The analysis was performed with 333,107 individuals of white British ancestry from the U.K. Biobank with complete information for the additional covariates, and among them, 317,737 individuals had available creatinine-based eGFR values.

## Data Availability

The data described in the manuscript will be made available from the U.K. Biobank consortium after acquiring approval (URL: https://biobank.ctsu.ox.ac.uk/crystal/docs.cgi?id=1, last accessed 7 March 2021). The code book and analytic code will be made available by the corresponding author upon reasonable request.
